# HUNK Phosphorylates Rubicon to Support Autophagy

**DOI:** 10.3390/ijms20225813

**Published:** 2019-11-19

**Authors:** Joelle N. Zambrano, Scott T. Eblen, Melissa Abt, J. Matthew Rhett, Robin Muise-Helmericks, Elizabeth S. Yeh

**Affiliations:** 1 Department of Cell and Molecular Pharmacology and Experimental Therapeutics, Medical University of South Carolina, Charleston, SC 29425, USA; joellezambrano@gmail.com (J.N.Z.); eblen@musc.edu (S.T.E.); rhettj@musc.edu (J.M.R.); 2 Department of Pharmacology and Toxicology, Indiana University School of Medicine, Simon Cancer Center, Indianapolis, IN 46202, USA; mabt@iu.edu; 3 Department of Regenerative Medicine and Cell Biology, Medical University of South Carolina, Charleston, SC 29425, USA; musehelm@musc.edu

**Keywords:** HUNK, phosphorylation, Rubicon, autophagy, kinase

## Abstract

Background: Autophagy is a catabolic cellular recycling pathway that is essential for maintaining intracellular homeostasis. Autophagosome formation is achieved via the coordination of the Beclin-1 protein complex. Rubicon is a Beclin-1 associated protein that suppresses autophagy by impairing the activity of the class III PI3K, Vps34. However, very little is known about the molecular mechanisms that regulate Rubicon function. Methods: In this study, co-immunoprecipitation and kinase assays were used to investigate the ability of Hormonally Upregulated Neu-associated Kinase (HUNK) to bind to and phosphorylate Rubicon. LC3B was monitored by immunofluorescence and immunoblotting to determine whether phosphorylation of Rubicon by HUNK controls the autophagy suppressive function of Rubicon. Results: Findings from this study identify Rubicon as a novel substrate of HUNK and show that phosphorylation of Rubicon inhibits its function, promoting autophagy.

## 1. Introduction

Macroautophagy (referred to as autophagy) is a catabolic cellular recycling pathway that is essential for maintaining intracellular homeostasis [1,2,3]. Coined for the Latin term “self-eating,” autophagy results in the digestion of cytoplasmic constituents that are degraded and subsequently eliminated by the cell, or recycled as biological precursors for the synthesis of new macromolecules [1,2,3]. Autophagy is initiated by the formation of a double membraned vesicle called the autophagosome, which is achieved via activation of the Beclin-1 protein complex [1,2,3,4]. As the autophagosome forms, it envelops cytoplasmic material that can range from proteins targeted for degradation to entire organelles. The engulfment of cytoplasmic material by the autophagosome can either be non-selective or selective. As the autophagosome matures, it fuses with a neighboring lysosome to form the autolysosome. Because the lysosome contains hydrolases, fusion of these two vesicles results in degradation of the cellular contents within the autolysosome. These degradation products are then removed by the cell via exocytosis or taken back up by the same cell to be used as starting material for the synthesis of new macromolecules [1,2,3].

Autophagosome maturation is regulated by a protein complex containing the essential autophagy protein Beclin-1, which binds to a core group of proteins comprising, but not limited to, the class III PI3 Kinase Vps34, Atg14L, UVRAG, and Rubicon [4,5,6,7,8]. UVRAG and Rubicon interact selectively to either positively or negatively regulate autophagosome formation and consequently regulate flux through the autophagy pathway. Kinases including AMPK, mTOR, and Akt are known regulators of autophagy and prior reports show that proteins within the Beclin-1 complex, including Beclin-1, are regulated by phosphorylation [9]. AMPK phosphorylates S93, S96, and T388 in Beclin-1 to enhance autophagy, whereas EGFR phosphorylates Beclin-1 at Y233 to inhibit autophagy [10,11,12]. The autophagy kinase ULK1 phosphorylates Vps34 to induce its lipid kinase activity, thereby enhancing autophagy [13]. Likewise, regulatory proteins upstream of the Beclin-1 complex are phosphorylated. ULK1 is phosphorylated by AMPK and mTOR, which have opposing functions in autophagy, where AMPK phosphorylates ULK1 at S317 and S777 to promote autophagy and mTOR phosphorylates ULK1 at S757 to inhibit autophagy [14].

We recently showed that the protein kinase, HUNK, regulates autophagy [15,16]; however, the mechanism by which HUNK carries out this function is unknown. In this study, we show that HUNK binds to the Beclin-1 protein complex and subsequently phosphorylates the autophagy regulatory protein Rubicon. Functionally, Rubicon suppresses autophagy by inhibiting Vps34 lipid kinase activity [17,18,19]. Since HUNK is a pro-autophagy protein and Rubicon inhibits autophagy when it is bound to the Beclin-1 complex, we determined whether phosphorylation of Rubicon by HUNK inhibits Rubicon function. We found that a phosphorylation deficient mutant of Rubicon suppresses Vps34 activity and inhibits autophagy, indicating that HUNK phosphorylation of Rubicon supports autophagy.

## 2. Results

### 2.1. HUNK Kinase Activity is Sufficient for Autophagy

**Figure 1:**
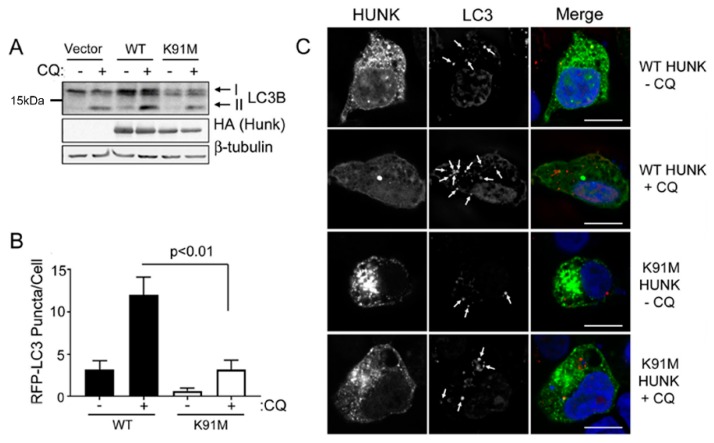
Hormonally Upregulated Neu-associated Kinase (HUNK) activity is required for HUNK-mediated autophagy. (**A**) 293T cells were transfected with empty vector, HA-HUNK, or HA-K91M HUNK. Cells were then serum deprived and treated with 100 µM CQ for 4 h, lysed and analyzed for LC3B by immunoblot analysis. (**B**) 293T cells plated on coverslips were transfected with RFP-LC3B and either GFP-HUNK or GFP-K91M-HUNK. Cells were serum deprived and treated with 100 µM CQ for 4 h, followed by fixation and imaging. Quantitation of RFP-LC3B puncta in cells. *N* ≥ 3 fields per experiment. Data represent 3 or more experiments. Student’s *T*-test was used to perform statistical analysis. (**C**) Representative images of quantitation in (**B**). Scale bar size = 10 µm.

Prior studies from our lab showed that HUNK promotes autophagy, but it is unknown if this function is dependent on HUNK’s enzymatic activity [15,16]. To address this question, we evaluated cells expressing an empty vector, wildtype (WT) HUNK or a kinase inactive (K91M) mutant of HUNK by immunoblotting for endogenous LC3B protein to assess LC3BII protein accumulation. WT HUNK-expressing cells showed an increase in LC3BII after 4 h of serum deprivation in the presence of the late-stage autophagy inhibitor chloroquine (CQ), compared to empty vector and K91M HUNK-expressing cells (Figure 1A). We also observed that levels of p62 were suppressed when WT HUNK was expressed in the absence of CQ but that empty vector and K91M HUNK-expressing cells had elevated levels of p62, regardless of CQ treatment (Supplementary Figure S1). Additionally, we evaluated LC3B puncta formation in cells expressing either WT HUNK, or K91M HUNK. 293T cells were transfected with RFP-LC3 and GFP-WT HUNK or GFP-K91M HUNK. Cells were serum deprived for 4 h, treated with CQ, and imaged by confocal microscopy to detect LC3B puncta, indicating the presence of autophagosomes. In basal and CQ-treated conditions, cells expressing WT HUNK had higher levels of LC3B-positive (LC3+) puncta than cells expressing K91M HUNK (Figure 1B,C). Altogether, these data suggest that HUNK kinase activity is required for HUNK-mediated autophagy.

### 2.2. HUNK Binds the Beclin-1 Protein Complex

**Figure 2:**
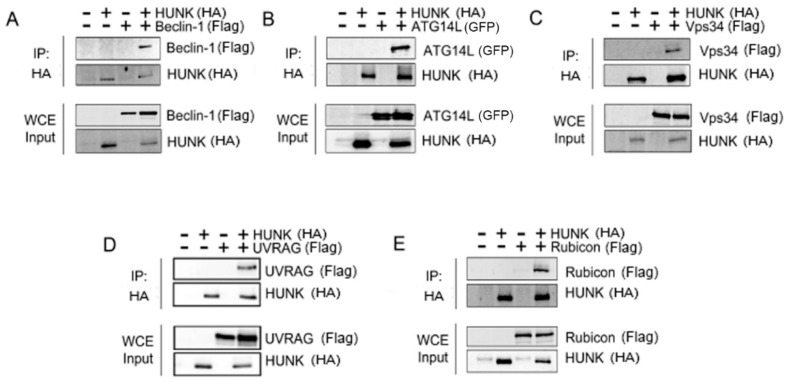
HUNK binds the Beclin-1 complex members. HA-HUNK and either (**A**) Flag-Beclin-1, (**B**) GFP-Atg14L, (**C**) Flag-Vps34, (**D**) Flag-UVRAG, or (**E**) Flag-Rubicon were co-transfected into 293T cells. HUNK was immunoprecipitated and binding of individual proteins was assessed via immunoblotting for each Beclin-1 complex member. Each member of the Beclin complex co-immunoprecipitated with HUNK. HUNK was immunoprecipitated and detected with anti-HA antibody and Beclin-1 complex members were detected with anti-Flag or anti-GFP antibodies. Interactions were also detected by individual antibodies to each Beclin-1 complex member.

Because the Beclin-1 complex is essential for autophagosome formation and maturation, we investigated whether HUNK binds to this complex. HUNK and Beclin-1 complex members were transfected into 293T cells and analyzed for protein–protein interactions via co-immunoprecipitation. Each complex member was transfected individually in conjunction with HUNK. HUNK co-immunoprecipitated with Beclin-1 (Figure 2A), Atg14 (Figure 2B), Vps34 (Figure 2C), UVRAG (Figure 2D), and Rubicon (Figure 2E), suggesting that HUNK is able to bind the Beclin-1 complex.

### 2.3. HUNK Phosphorylates Rubicon within the N-terminus of the Protein

**Figure 3:**
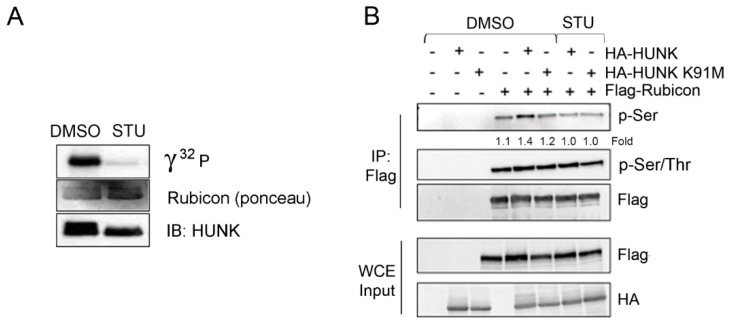
HUNK phosphorylates Rubicon (**A**) In vitro HUNK kinase assay using recombinant Rubicon (aa 1-375) as substrate. HUNK was preincubated with either DMSO or the HUNK inhibitor staurosporine (STU, 5 µM). (**B**) HA-HUNK or HA-K91M HUNK and Flag-Rubicon were expressed in 293T cells. Flag-Rubicon was immunoprecipitated using anti-Flag affinity resin and isolated protein was immunoblotted using anti-pSer and anti-pSer/Thr antibodies.

Since HUNK kinase activity is required to promote autophagy and HUNK binds to protein in the Beclin-1 complex, we investigated whether HUNK could phosphorylate these proteins. Using in vitro HUNK kinase assays, we found that HUNK directly phosphorylates Rubicon (Figure 3A) as well as Beclin-1 and Atg14L (Supplementary Figure S2) and that pre-treatment of the reactions with the HUNK inhibitor staurosporine (STU) [20], inhibited phosphorylation of each individual substrate.

Although Rubicon was identified a decade ago [18,19], little is known about the molecular mechanisms that regulate its function. Consequently, we further investigated HUNK-directed phosphorylation of Rubicon and the role of this event in regulating autophagy. HUNK is a serine/threonine kinase and there are 45 predicted serine or threonine residues in Rubicon that could be phosphorylated by HUNK according to data from PhosphoSitePlus^®^. To narrow down the potential site(s) of phosphorylation, we first addressed whether HUNK phosphorylated serine and/or threonine residues within Rubicon. We immunoprecipitated Flag-Rubicon from cells co-transfected with either WT HUNK or K91M HUNK and probed for phosphorylation of Rubicon using phospho-serine (pSer) and phospho-serine/threonine (pSer/Thr) antibodies. Rubicon isolated from cells containing WT HUNK showed an increase in pSer (~1.3 fold, *p* = 0.02) compared to Rubicon from cells expressing Rubicon alone (Figure 3B). This increase in pSer Rubicon was ablated when Rubicon was isolated from cells expressing K91M HUNK or expressing WT HUNK in the presence of the HUNK kinase inhibitor STU (Figure 3B). These changes in phosphorylation were not seen when probing with the pSer/Thr antibody, suggesting that the increase in Rubicon phosphorylation in the presence of HUNK is predominantly due to HUNK phosphorylation of Rubicon on one or more serine residues. We next used 293T cells engineered with CRISPR/Cas9 to target HUNK to determine whether loss of HUNK impaired Rubicon phosphorylation (Supplementary Figure S3A) [21]. Flag-Rubicon was expressed in control and HUNK CRISPR knockout 293T cells, isolated by immunoprecipitated and probed for pSer. We detected pSer on Flag-Rubicon isolated from control cell but not the HUNK-depleted cells (Supplementary Figure S3B).

**Figure 4:**
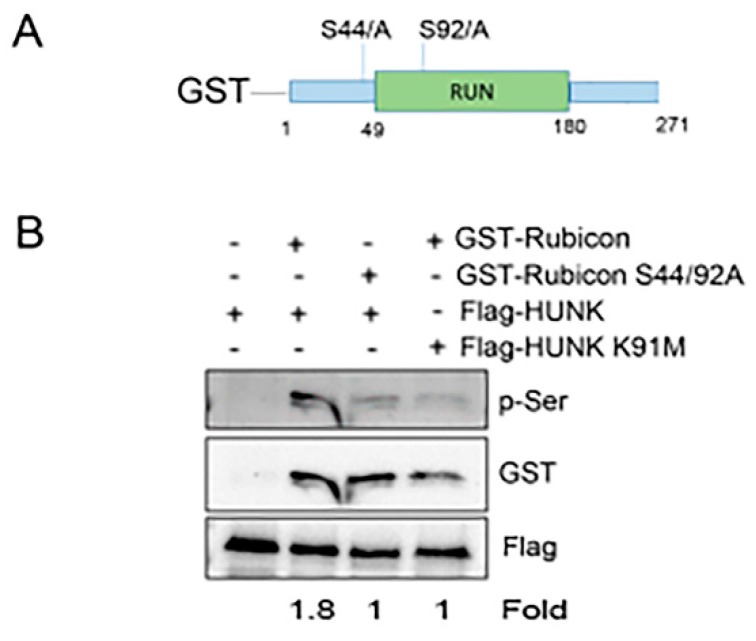
HUNK phosphorylates the N-terminal region of Rubicon. (**A**) GST-Rubicon containing amino acids 1-271 with S44 and S92 mutated to alanine (**B**) In vitro kinase assay using Flag-WT HUNK and Flag-K91M HUNK as kinase and GST-Rubicon or GST-Rubicon S44/92A as substrate. Reactions were immunoblotted for p-Ser to detect Rubicon phosphorylation and GST or Flag to confirm the presence of HUNK and Rubicon in each reaction.

The recombinant form of Rubicon protein that we used in Figure 3A only included amino acids 1-375 (aa 1-375), demonstrating that HUNK phosphorylated the N-terminus of Rubicon, although not ruling out additional sites of phosphorylation C-terminal to aa 375. The N-terminus of Rubicon contains a unique region called the RUN domain, a protein binding domain typically found in Rab proteins. The RUN domain of Rubicon was previously shown to be required for Rubicon’s capacity to suppress autophagy [17]. There are only two serine residues that lie either within the RUN domain (i.e., serine (S) 92) or within 10 amino acids of the RUN domain (i.e., S44). Therefore, we constructed a GST-tagged truncated version of WT or S44 and S92 mutated to alanine (A) Rubicon containing amino acids 1-271 (Figure 4A) and isolated recombinant protein to use as substrate for a HUNK kinase assay. Flag-WT HUNK and Flag-K91M HUNK were expressed in 293T cells and isolated for use in an in vitro kinase assay with GST-Rubicon and GST-S44/92A Rubicon as substrates. Kinase reactions were probed with anti-pSer antibody to assess GST-Rubicon phosphorylation by HUNK. Our results showed that HUNK phosphorylated GST-WT Rubicon, but that HUNK did not phosphorylate GST-S44/92A Rubicon (Figure 4B).

### 2.4. HUNK Phosphorylation of Rubicon Promotes Autophagosome Formation

**Figure 5:**
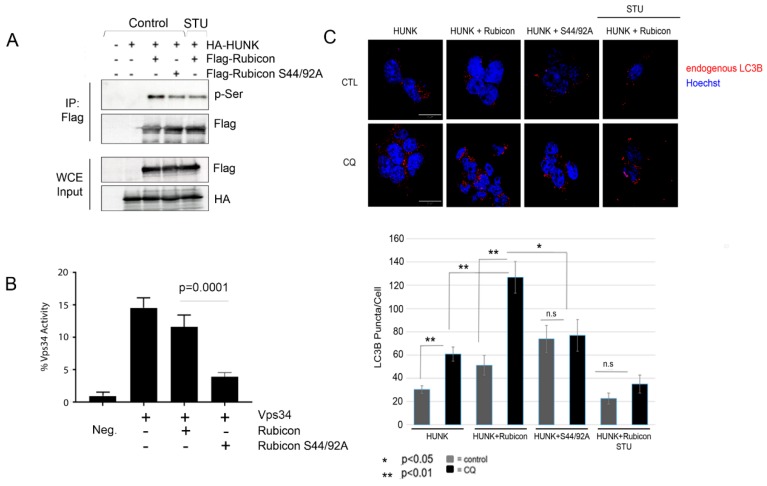
Autophagy is impaired in cells expressing WT HUNK and S44A/S92A Rubicon. (**A**) HA-HUNK and Flag-Rubicon WT or Flag-Rubicon S44A/S92A were expressed in 293T cells. The next day, cells were treated with either vehicle (DMSO-Control group) or 100 nM STU for 1 h prior to lysing. Rubicon was isolated by Flag immunoprecipitation and immunoblotted for p-Ser and Flag. Whole cell extracts (WCE) were immunblotted for Flag and HA to confirm HUNK and Rubicon expression. (**B**) Flag-Vps34 was expressed with WT Rubicon or S44/92A Rubicon. Vps34 was isolated by Flag immunoprecipitation and incubated with PI substrate in a kinase reaction. Percent Vps34 activity is a quantitation of PI to PI(3)P conversion. Data represents 3 individual experiments. Student’s *T*-test was performed on *n* = 6 replicates, *p* = 0.0001. (**C**) HA-HUNK was expressed with WT Rubicon or S44A/S92A Rubicon in 293T cells. The following day cells were serum deprived and treated with 100 µM chloroquine (CQ) for 4 h and then (DMSO) or 100 nM STU for 1 h prior to imaging. Representative images shown (top) with quantitation of the number of endogenous LC3B puncta per cell (bottom). *N* ≥ 3 fields per experiment. Data represent 3 or more experiments. Student’s *T*-test was used to perform statistical analysis. Scale bar size = 10 µm, not significant (*n.s.)*.

Because HUNK phosphorylates Rubicon in the N-terminus where the RUN domain is located, we hypothesized that this phosphorylation event inhibits Rubicon activity and induces autophagy. Therefore, we generated a full-length form of the phospho-deficient mutant form of Rubicon (S44/S92A Rubicon) and confirmed that the S44/92A Rubicon was phosphorylation deficient by expressing S44/S92A Rubicon in the presence of WT HUNK in 293T cells. Rubicon or S44/92A Rubicon were then isolated by immunoprecipitation and assessed for levels of phosphorylation by immunoblotting with a pSer antibody. We observed a decrease in pSer with the S44/92A Rubicon mutant compared to WT Rubicon isolated from cells expressing WT HUNK (Figure 5A). We also observed that the level of phosphorylation seen with S44/92A Rubicon was similar to the level seen when WT Rubicon was isolated from cells that were treated with STU to suppress HUNK kinase activity (Figure 5A). Since the RUN domain of Rubicon was previously reported to be important for Vps34 binding, we also looked at binding between HUNK, Beclin-1, and Vps34 in the presence of WT Rubicon or S44/92A Rubicon and saw no change in levels of interaction between these proteins (Supplementary Figure S4).

We next assessed the effect of the S44/92A Rubicon phosphorylation deficient mutant toward Vps34 kinase activity. Flag-Vps34 was expressed in 293T cells in the presence of full-length Flag-WT Rubicon or the Flag-S44/92A Rubicon mutant. Vps34 was isolated by Flag immunoprecipitation and used in a kinase assay to measure phosphotidylinositol (PI) conversion to phosphatidylinositol 3-phosphate (PI(3)P). We found that when S44/92A Rubicon was associated with Vps34, there was reduced PI to PI(3)P conversion levels compared to Vps34 bound to WT Rubicon (Figure 5B).

To assess autophagy, we expressed HUNK in conjunction with WT Rubicon or S44/92A Rubicon in 293T cells and monitored for endogenous LC3B puncta formation in the presence or absence of CQ treatment after 4 h of serum deprivation. We also used STU as a control to inhibit HUNK kinase activity. We found that co-expression of WT Rubicon and WT HUNK showed an increase in LC3B positive puncta under CQ-treated conditions compared to WT HUNK alone (Figure 5C). Co-expression of WT HUNK and Rubicon S44A/S92A did not show an increase in autophagy beyond the level seen with HUNK alone, regardless of CQ treatment (Figure 5C). Consistent with these observations, WT HUNK and Rubicon expressed in cells that were treated with STU showed reduced levels of autophagy (Figure 5C). Taken together, this set of data suggests that HUNK phosphorylates Rubicon in the N-terminus where the RUN domain region containing amino acids S44 and S92 is located and that the consequence of these phosphorylation events is to relieve the suppressive effect that Rubicon has on autophagy through regulation of Vps34 activity.

## 3. Discussion

In this study, we show that HUNK-mediated regulation of autophagy is dependent on HUNK kinase activity. We also show that HUNK binds several members of Beclin-1 complex, a key regulator of autophagy. Our kinase assay results identify Rubicon as a novel HUNK substrate. Rubicon contains a RUN domain in its N-terminus that is required for Rubicon to bind and suppress Vps34 [17]. In this study, we hypothesized that HUNK phosphorylated Rubicon in or near the RUN domain since this domain is necessary for Rubicon-directed suppression of autophagy. Using a HUNK phosphorylation deficient Rubicon mutant (S44A/S92A Rubicon), we found that expression of this mutant with WT HUNK resulted in an impairment of autophagy, whereas expression of WT Rubicon with WT HUNK did not. We also found that Vps34 kinase activity is suppressed by S44/92A Rubicon. Based on these observations, we concluded that HUNK phosphorylation of Rubicon inhibits the suppression of autophagy by Rubicon in a Vps34-dependent manner. These findings are consistent with our prior findings, that HUNK promotes autophagy and provide a molecular mechanism for those prior findings [15,16].

While these results indicate that HUNK phosphorylates Rubicon’s N-terminal region, this does not exclude the possibility that HUNK phosphorylates other sites in Rubicon. Rubicon contains many serine residues close together, particularly in the middle of the protein, and due to limitations in isolating full-length recombinant Rubicon, a full-length Rubicon protein was not tested for phosphorylation by HUNK kinase assay in the present study. Furthermore, although outside the scope of the present studies, we also found that HUNK phosphorylates Beclin-1 and Atg14L. Interestingly, we observed in Figure 5C that the levels of LC3B puncta were higher in samples containing WT HUNK and WT Rubicon as well as WT HUNK and S44/92A Rubicon, in the absence of CQ treatment. We attribute this effect to endogenous HUNK and Rubicon, and possibly Beclin-1 and Atg14L, which are likely enriched due to overexpression of these autophagy related protein and remain available for HUNK to phosphorylate. Therefore, further examination of these proteins as functional substrates of HUNK have the potential to show that HUNK regulates autophagy at multiple levels within the Beclin-1 complex.

In addition to suppressing autophagy, Rubicon also functions as a negative regulator of endocytosis. Rubicon prevents endosome maturation by binding to UVRAG and the Rab-GTP protein Rab7. When Rubicon is bound to UVRAG, it is sequestered away from the class C-VPS/HOPS complex and Rab7 is maintained in an inactive, GDP-bound state [22]. GTP-bound Rab7 competes with UVRAG for Rubicon binding to release sequestered UVRAG, thereby promoting endosomal maturation [22,23]. Whether HUNK binding to Rubicon regulates the interaction between Rubicon and UVRAG or Rab7 is unknown and will need to be explored further.

Prior findings suggest that HUNK promotes autophagy as a mechanism for acquiring resistance to HER2 targeted inhibitors in HER2-positive breast cancer cells [15,16]. However, it is not known whether HUNK’s kinase activity is required for the development of resistance in the context of Rubicon regulation. Consequently, future studies will be required to address whether phosphorylation of Rubicon by HUNK supports the development of resistance in breast cancer. Taken together, our findings are the first to demonstrate that HUNK regulates autophagy through the direct phosphorylation of the autophagy regulatory protein Rubicon.

## 4. Materials and Methods

### 4.1. Cell Culture

Cells were kept in a humidified incubator at 37 °C and 5% CO_2_. 293T cells were grown in DMEM (Corning, New York, USA) supplemented with 10% fetal bovine serum (FBS, Gibco, ThermoFisher Scientific, Waltham, MA, USA). pcDNA_6_-GFP-Rubicon-Flag was a gift from Qing Zhong (Addgene plasmid #28022; http://n2t.net/addgene:28022; RRID:Addgene_28022). Flag-HUNK, HA-HUNK, and EGFP-HUNK were a gift from Lewis Chodosh (University of Pennsylvania, Philadelphia, PA, USA). pmRFP-LC3 was a gift from Tamotsu Yoshimori (Addgene plasmid #21075; http://n2t.net/addgene:21075; RRID:Addgene_21075). p3xFLAG-CMV10-hUVRAG was a gift from Noboru Mizushima (Addgene plasmid # 24292; http://n2t.net/addgene:24292; RRID:Addgene_24292). pEGFP-Atg14L was a gift from Tamotsu Yoshimori (Addgene plasmid #21635; http://n2t.net/addgene:21635; RRID:Addgene_21635). pcDNA_4_-Vps34-Flag was a gift from Qing Zhong (Addgene plasmid #24398; http://n2t.net/addgene:24398; RRID:Addgene_24398). pcDNA_4_-Beclin1-Flag (FL) was a gift from Qing Zhong (Addgene plasmid #24388; http://n2t.net/addgene:24388; RRID:Addgene_24388).

### 4.2. Immunoblotting

All cells were lysed in buffer containing 50 mM Tris-HCl, pH 7.5, 150 mM sodium chloride, 1 mM EDTA, 1% Triton X-100 with HALT protease and phosphatase inhibitor cocktail (Thermo Scientific, Waltham, MA, USA). Primary antibodies used for Western blotting include: anti-Flag-M2 (Sigma-Aldrich, St. Louis, MO, USA, #F1804), anti-HUNK (Invitrogen, ThermoFisher Scientific, Waltham, MA, USA, PA5-28765), anti-HA (Cell Signaling, Danvers, MA, USA, #3724), anti-GFP (Santa Cruz, CA, USA, #sc-8334), anti-LC3B (Cell Signaling, #2775), anti-Rubicon (Cell Signaling, #D9F7), anti-phosphoserine (Abcam, Cambridge, UK #9332), anti-phosphoserine/threonine (Abcam, #17464), anti-HA (Cell Signaling, #C29F4), anti-GST (Santa Cruz, sc-53909), anti-Beclin-1 (Santa Cruz, sc-48381), anti-UVRAG (ThermoFisher, #23215), anti-Atg14L (Cell Signaling, #5504), anti-PI3K class III (aka Vps34, Cell Signaling, #4263), and anti-β-tubulin (Santa Cruz, sc-55529). Imaging was performed on the FluorChem-R imaging system and quantitated using Alpha View SA software (Protein Simple, San Jose, CA, USA, version 3.4.0).

### 4.3. Immunofluorescence

For RFP-LC3 imaging, equal numbers of 293T cells were plated on gelatin-coated coverslips (Corning). The next day, cells were transfected with either EGFP-HUNK or EGFP-K91M HUNK and pmRFP-LC3B using polyethylenimine (PEI). After 24 h, cells were treated with 100 µM chloroquine (CQ) for 4 h, followed by fixation with 4% paraformaldehyde. For endogenous LC3B analysis, 293T cells were plated and transfected with pcDNA_3_-HA-HUNK and either pcDNA_6_-GFP-Rubicon-Flag or pcDNA_6_-GFP-Rubicon S44A/S92A-Flag using PEI. Cells were then pre-treated with 100 nM staurosporine (STU) or DMSO for 1 h, followed by treatment with 100 µM CQ for 4 h. Cells were then fixed in 4% paraformaldehyde, followed by permeabilization with 0.1% Triton X-100 in PBS. Anti-LC3B (Cell Signaling, #2775) and goat-anti-rabbit Alexa Fluor 594 (Life Technologies, ThermoFisher Scientific, Waltham, MA, USA) antibodies were used for staining. Imaging was performed using a Leica DMi8 confocal microscope.

### 4.4. Immunoprecipitation Assay

Equal numbers of 293T cells were plated and transfected the next day with HA-HUNK, GFP-Atg14L, Flag-Beclin-1, Flag-UVRAG, Flag-Rubicon, and Flag-Vps34. Equal protein was used to immunoprecipitate HUNK from cell lysates using HA or Flag antibody coupled to resin (protein A (Biorad, Des Plaines, IL, USA), protein G sepharose (Biorad), Flag resin (Sigma)), in lysis buffer and protease/phosphatase inhibitors. Lysates rotated overnight at 4°C and the next day beads were washed 3–4 times in lysis buffer prior to being prepared for immunoblotting analysis.

### 4.5. HUNK Kinase Assay

Recombinant HUNK protein was purchased from ThermoFisher (Invitrogen, #A30974). For kinase reactions, the kinase was incubated in kinase buffer (20 mM HEPES, pH 7.3 and 2 mM MgCl), 100 µM cold ATP, 10 µCi of γ-32P-ATP (PerkinElmer) and substrate: human Rubicon protein (Novus Biologicals, H00009711); Beclin-1 (Ray Biotech, #228-21395-2); UVRAG (MyBioSource, MBS1365072); and Atg14L (MyBioSource, MBS1449868). All kinase reactions were incubated at 30 °C for 20 min. For GST-Rubicon kinase assays, Flag-HUNK was expressed in 293T cells and isolated using anti-Flag affinity beads (Sigma, M8823). Kinase was eluted using Flag peptide (Sigma, F3290). Eluted kinase was mixed with GST-Rubicon and GST-S44/92A Rubicon and incubated in kinase buffer (20 mM HEPES, pH 7.3 and 2 mM MgCl) and 1 mM ATP. Kinase reactions were incubated at 30 °C for 20 min. Anti-phosphoserine antibody (Abcam, #9332) was used to detect Rubicon phosphorylation. GST-Rubicon and GST-S44/92A Rubicon were generated by cloning amino acids 1-271 of Rubicon into pGEX6P. pcDNA_6_-GFP-Rubicon-Flag was used to PCR amplify amino acids 1-271 of Rubicon. PCR primers: Forward primer: 5′ CATGGTCCTGCTGGAGTTCGTG 3′, Reverse primer: 5′ GAATTCTTTAATCATTGATCCTCTGC 3′. PCR product was digested with BamHI and EcoRI and ligated into pGEX6P.

### 4.6. Vps34 Kinase Assay

Vps34 activity was measured by kinase assay detected with competitive ELISA (Echelon Biosciences, K3000). Flag-Vps34 was isolated from lysates by immunoprecipitation (IP) using anti-Flag affinity beads and incubated in kinase reaction buffer containing 20 mM Tris pH 8.0, 200 mM NaCl, 2 mM EDTA, 100 µM ATP, and 20 mM MnCl_2_ and PI substrate. Reaction were stopped and added to a PI(3)P-coated microplate for competitive binding to a PI(3) detector protein. The amount of PI(3)P detector bound to the plate was determined by colorimetric detection. Signal is inversely proportional to the amount of PI(3)P produced.

## 5. Conclusions

Findings from this study demonstrate that HUNK phosphorylates the N-terminus of Rubicon and that loss of Rubicon phosphorylation by HUNK is sufficient to inhibit Vps34 activity. The net effect of Rubicon phosphorylation by HUNK is to inhibit Rubicon function and support autophagy.

## Supplementary Materials

Supplementary materials can be found at https://www.mdpi.com/1422-0067/20/22/5813/s1.

## Abbreviations

Beclin-1	Coiled-coil myosin-like BCL2-interacting protein
Vps34	Vacuolar protein sorting, class III PI 3-kinase
Atg14L	Autophagy related 14 like
UVRAG	UV radiation resistance-associated gene protein
Rubicon	RUN domain Beclin-1-interacting and cysteine-rich domain-containing protein
AMPK	AMP-activated protein kinase
mTOR	Mammalian target of rapamycin
Akt	Protein kinase B
ULK1	Unc-51 like autophagy activating kinase 1
EGFR	Epidermal growth factor receptor
HUNK	Hormonally upregulated neu-associated kinase
LC3B	Microtubule-associated proteins 1A/1B light chain 3B
WT	Wildtype
RFP	Red fluorescent protein
GFP	Green fluorescent protein
CQ	Chloroquine
RUN	RPIP8, unc-14, and NESCA domain
STU	Staurosporine
GST	Glutathione S-transferase
DMSO	Dimethyl sulfoxide
Rab7	Ras-related protein 7
GDP	Guanosine diphosphate
GTP	Guanosine triphosphate
HER2	Human epidermal growth factor receptor 2
