# Supplementary material for: HUNK Phosphorylates Rubicon to Support Autophagy
Source: Int J Mol Sci. 2019 Nov 19;20(22):5813. doi: 10.3390/ijms20225813 (PMC6888122; doi:10.3390/ijms20225813)
Supplement: Supplementary file 1 [file ijms-20-05813-s001.pdf]

Supplementary Figures

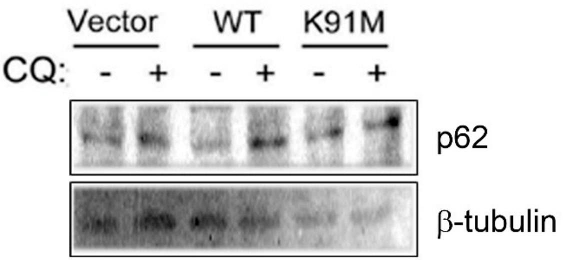

Supplementary Figure 1. HUNK expression suppress p62 levels in the absence of chloroquine. 293T cells were transfected with empty vector, HA-HUNK, or HA-K91M HUNK (shown in Figure 1). Cells were then treated with 100 µM CQ for 4 hours lysed and analyzed by immunoblot analysis. Here, we probed for p62 expression using anti-p62 antibody.

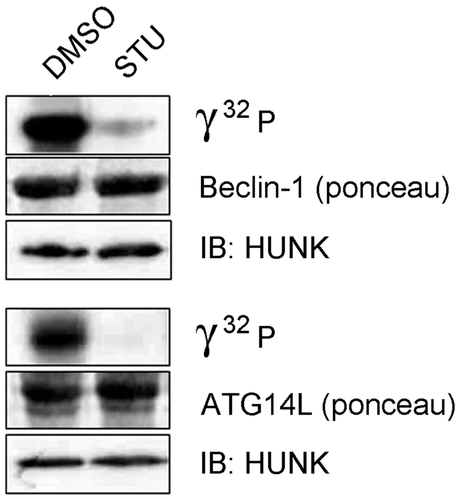

Supplementary Figure 2. HUNK phosphorylates Beclin-1 and ATG14L. In vitro HUNK kinase assay using recombinant Beclin-1 or ATG14L as substrate. HUNK was preincubated with either DMSO or the HUNK inhibitor staurosporine (STU, 5 µM).

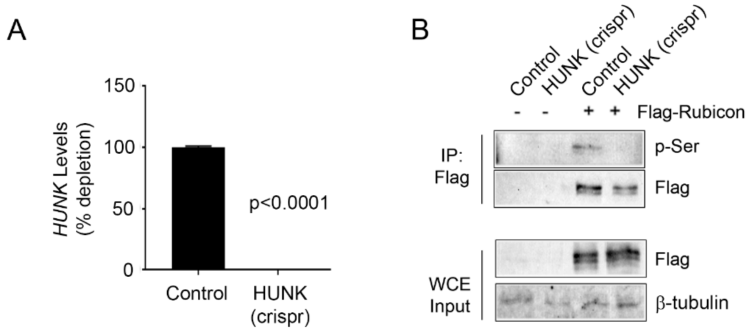

Supplementary Figure 3. Phospho-serine Rubicon is reduced in HUNK depleted cells. (A) Quantitative RealTime PCR was used to confirm depletion of *HUNK* in 293T cells after sgRNA targeting using CRISPR/Cas9. **(B)** Flag-Rubicon was expressed in control or HUNK CRISPR depleted 293T cells and isolated using anti-Flag affinity resin. Isolated Flag-Rubicon was immunoblotted using anti-pSer and anti-Flag antibodies.

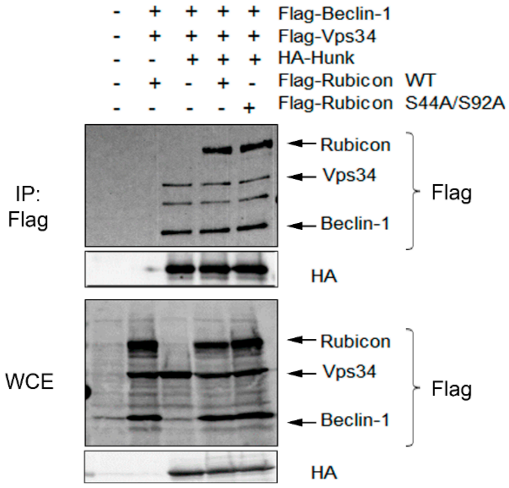

Supplementary Figure 4. S44/92A Rubicon does not disrupt the interaction between HUNK, Beclin-1, and Vps34. HA-HUNK was co-expressed in 293T cells with Beclin-1, Vps34, WT Rubicon, or S44/92A Rubicon as indicated. HUNK was immunoprecipitated with anti-HA antibody and probed for Flag to detect Beclin-1, Vps34, and Rubicon.
